# Characterization of the complete chloroplast genome of *Biondia chinensis* (Apocynaceae: Asclepiadoideae: Asclepiadeae), a rare and threatened liana endemic to China

**DOI:** 10.1080/23802359.2018.1491340

**Published:** 2018-07-11

**Authors:** Hua Rao, Xiao-Juan Wang, Jian-Xiang Ma, Qi-En Li, Guang-Lin Li, Jia-Bin Zou

**Affiliations:** a College of Life Sciences, Shaanxi Normal University, Xi’an, People’s Republic of China;; b MOE Key Laboratory of Bio-Resources and Eco-Environment, College of Life Science, Sichuan University, Chengdu, People’s Republic of China;; c Tibetan Medicine Research Center of Qinghai University, Qinghai University Tibetan Medical College, Xining, People’s Republic of China

**Keywords:** Complete chloroplast genome, Biondia chinensis, genome assembly, phylogeny

## Abstract

The complete chloroplast genome of *Biondia chinensis*, a rare liana of the Asclepiadoideae endemic to China, was determined in this study. It is classified as Vulnerable species because of the sharp decline in its population size due to the habitat destruction. The whole chloroplast genome was 160,308 bp long, comprising of a large single copy (LSC) region of 91,335 bp and a small single copy (SSC) region of 19,185 bp, which were separated by a pair of 24,894 bp long inverted repeat (IR) regions. It encoded a total of 131 genes, including 86 protein-coding genes, 37 tRNA genes, and eight rRNA genes. Most of the gene species appeared as a single copy, while 22 gene species appeared in double copies. The overall A + T content was 62.2%, while the corresponding values of the LSC, SSC, and IR regions were 63.9, 68.1, and 56.7%, respectively. Phylogenetic analysis suggested that, among all the species which have been analyzed *B. chinensis* was relatively close to *Vincetoxicum rossisum*.

Biondia chinensis, a species of the subfamily Asclepiadoideae of the family Apocynaceae, is an endemic liana mainly distributed in south-western China and found in the mountain forests, roadsides, and bottom of the cliffs at altitudes of 1000–2000 m (Li et al. 1995). This plant not only has a great potential in creating vertical greening landscape in the cities (Ying et al. 1993), but also can be used as a traditional Chinese medicine for the treatment of traumatic injuries (Tan et al. 2003). However, it has been classified as Vulnerable in IUCN Red List of Threatened Species since 2006 (IUCN 2017), mainly because of its sharp decline in species diversity and population size due to a very limited distribution and habitat destruction. In addition, B. chinensisis is one of the representative species of the Biondia, this genus is known to be a small satellite genus within Asclepiadeae family and shows a close relationship with Vincetoxicum and/or Tylophora (Liedeschumann et al. 2012). However, the problem of its taxonomical phylogenetical position is still doubtful because of the lack of genomic information. In the present study, the complete chloroplast genome sequence of *B. chinensis* is reported to contribute in conservation of this species and provide significant information for the complex taxon of the Asclepiadeae.

Genomic DNA was extracted from the fresh leaves of a specimen of *B. chinensis* collected from Qinling Mountain (109°55'12"E, 33°11'24"N; it was deposited at Shaanxi Normal University; accession number: ZJB-2017-152-1). Complete chloroplast genome of *B. chinensis* was sequenced on an Illumina Hiseq X Ten platform. The high quality reads were assembled into a complete chloroplast genome using Velvet (Zerbino and Birney 2008), with *Asclepias syriaca* (GenBank: KF386166.1) as the reference. The complete chloroplast genome was annotated using Geneious (Kearse et al. 2012; Wang et al. 2017) and was then submitted to GenBank (accession no. MH210646).

The size of chloroplast genome of *B. chinensis* was 160,308 bp, including a large single copy (LSC, 91,335 bp) region, a small single copy (SSC, 19,185 bp) region and two inverted repeat (IR, 24,894 bp) regions. The circular genome contained 131 genes, including 86 protein-coding genes (78 PCG species), eight rRNA genes (4 rRNA species) and 37 tRNA genes (27 tRNA species). Most of the gene species occurred in a single copy, while 22 gene species occurred in double copies, including four rRNA species (4.5S, 5S, 16S and 23S rRNA), 10 tRNA species and 8 PCG species. The overall A+T content was 62.2%, while the corresponding values of the LSC, SSC, and IR regions were 63.9, 68.1, and 56.7%, respectively.

A neighbor-joining tree (Figure 1) was constructed based on the complete chloroplast genome sequences of *B. chinensis* and other species from Apocynaceae using the program MEGA6 (Tamura et al. 2013). The result showed that *B. chinensis* was closely related to *Vincetoxicum rossisum*, and these two species were then clustered into a clade with other 15 Asclepiadeae species, which was consistent with the phylogenic study suggested by Liedeschumann et al. (2012).

**Figure 1.**
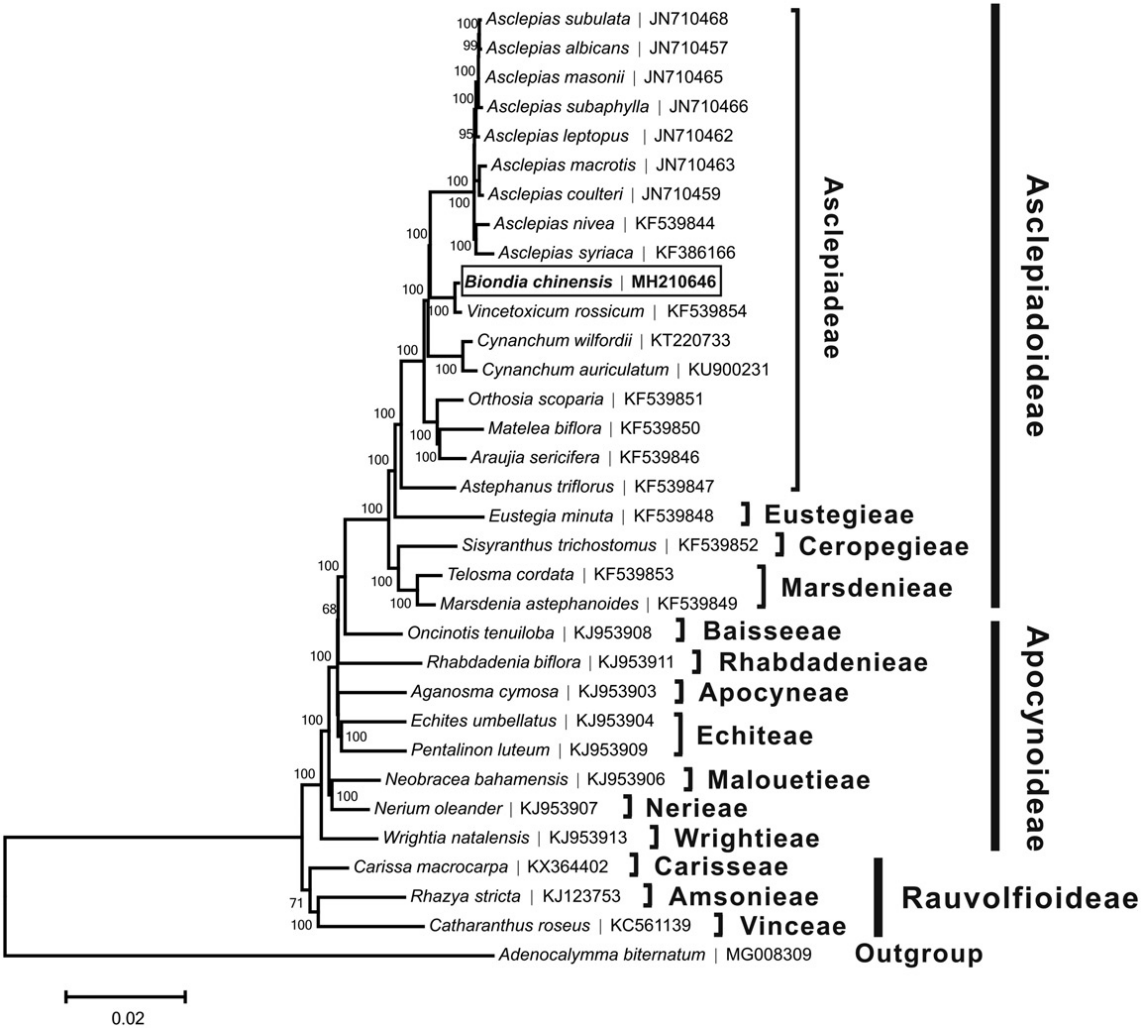
Neighbor-joining tree based on the complete chloroplast genome sequences of Biondia chinensis and related taxa within the Apocynaceae. The numbers on the branches are bootstrap values. The accession number of GenBank for each species is list in figure.
